# Taxonomic revision of Russula subsection Amoeninae from South Korea

**DOI:** 10.3897/mycokeys.75.53673

**Published:** 2020-11-09

**Authors:** Komsit Wisitrassameewong, Myung Soo Park, Hyun Lee, Aniket Ghosh, Kanad Das, Bart Buyck, Brian P. Looney, Miroslav Caboň, Slavomír Adamčík, Changmu Kim, Chang Sun Kim, Young Woon Lim

**Affiliations:** 1 School of Biological Sciences and Institute of Microbiology, Seoul National University, Seoul 08826, South Korea Seoul National University Seoul South Korea; 2 National Biobank of Thailand (NBT), National Science and Technology Development Agency (NSTDA), Thailand Science Park, Thanon Phahonyothin, Tambon Khlong Neung, Amphoe Klong Luang, Pathum Thani 12120, Thailand National Biobank of Thailand Pathum Thani Thailand; 3 Department of Botany & Microbiology, H.N.B. Garhwal University (A Central University), Srinagar, Garhwal, 246174, Uttarakhand, India Korea National Arboretum Pocheon South Korea; 4 A.J.C. Bose Indian Botanic Garden, Botanical Survey of India, P.O. Botanic Garden, Howrah 711103, India Garhwal University Srinagar India; 5 ISYEB (CNRS, Sorbonne Université, EPHE) Institut de Systématique, Évolution, Biodiversité, Muséum national d’Histoire naturelle, case postale 39, 57 rue Cuvier, F-75231 Paris cedex 05, France A.J.C. Bose Indian Botanic Garden Howrah India; 6 Department of Biology, Duke University, Durham, NC 27708, USA Muséum national d’Histoire naturelle Paris France; 7 Plant Science and Biodiversity Centre, Slovak Academy of Sciences, Dúbravská cesta 9, SK-845 23, Bratislava, Slovakia Duke University Durham United States of America; 8 Microorganism Resources Division, National Institute of Biological Resources, Incheon 22689, South Korea Slovak Academy of Sciences Bratislava Slovakia; 9 Forest Biodiversity Division, Korea National Arboretum, Pocheon-si, Gyeonggi-do 11186, South Korea National Institute of Biological Resources Incheon South Korea

**Keywords:** *
Amoeninae
*, *
Heterophyllae
*, multilocus phylogeny, *Russula orientipurpurea*, species delimitation

## Abstract

Russula subsection Amoeninae is morphologically defined by a dry velvety pileus surface, a complete absence of cystidia with heteromorphous contents in all tissues, and spores without amyloid suprahilar spot. Thirty-four species within subsection Amoeninae have been published worldwide. Although most *Russula* species in South Korea have been assigned European or North American names, recent molecular studies have shown that *Russula* species from different continents are not conspecific. Therefore, the present study aims to: 1) define which species of Russula subsection Amoeninae occur on each continent using molecular phylogenetic analyses; 2) revise the taxonomy of Korean *Amoeninae*. The phylogenetic analyses using the internal transcribed spacer (ITS) and multilocus sequences showed that subsection Amoeninae is monophyletic within subgenus Heterophyllidiae section Heterophyllae. A total of 21 Russula subsection Amoeninae species were confirmed from Asia, Australia, Europe, North America, and Central America, and species from different continents formed separate clades. Three species were recognized from South Korea and were clearly separated from the European and North American species. These species are *R. bella*, also reported from Japan, a new species described herein, *Russula orientipurpurea*, and a new species undescribed due to insufficient material.

## Introduction

*Russula* Pers. is the largest genus in the family Russulaceae, with at least 2,000 described species worldwide (Adamčík et al. 2019). Compared to most other genera of Basidiomycetes, *Russula* has complex morphological and chemical features (Buyck et al. 2018). The initial period of diversification of the genus has been inferred as occurring in temperate regions of the Northern hemisphere, though there is some debate as to its origin given its high diversity in tropical areas (Looney et al. 2016; Buyck et al. 2018). Recent molecular studies have recognized eight subgenera within the genus: Russula subg. Archaeae Buyck & V. Hofst., R. subg. Brevipedum Buyck & V. Hofst., R. subg. Compactae (Fr.) Bon, R. subg. Crassotunicatae Buyck & V. Hofst., R. subg. Glutinosae Buyck & X.H. Wang, R. subg. Heterophyllidiae Romagnesi, R. subg. Malodorae Buyck & V. Hofst., and R. subg. Russula (Buyck et al. 2018; Buyck et al. 2020).

In the field, specimens of Russula subsection Amoeninae Buyck are identified by their velvety pileus surface and stipe, white spore print, mild taste, and stipe flushed with pink, red, or purple. Microscopically, species of *Amoeninae* display subglobose spores, typically with prominent amyloid and reticulate ornamentation but without amyloid suprahilar spot. Moreover, the basidiomes completely lack gloeocystidia, and both pileipellis and lamellar edges have large subulate hyphal terminations often arising from short unbranched cells (Buyck 1988, 1994). Because of the complete absence of gloeocystidia, Sarnari (1998) altered the rank of this subsection to subgenus, proposing Russula subg. Amoenula Sarnari. Recent multilocus phylogenetic studies, however, have shown that *Amoeninae* is in fact a small part of R. subg. Heterophyllidiae section Heterophyllae (see position of *R. violeipes* Quél. and *R. mariae* Peck in Buyck et al. 2018).

To date, 34 species have been published worldwide within Russula subsection Amoeninae (Suppl. material 1: Table S1), some of which were not originally placed in this group (e.g. *R. diversicolor* Pegler, *R. epitheliosa* Singer, and *R. variegata* Romagn.). However, their microscopic features are very similar to those of other *Amoeninae* species. Three species have been reported from Europe: *R. amoena* Quél., *R. amoenicolor* Romagn., and *R. violeipes* Quél. (Sarnari 1998). Although no relevant molecular studies have been performed that include species from North America, previous studies using morphological data have listed 14 species for this area (Suppl. material 1: Table S1). Regarding the tropics, *R. diversicolor* and *R. epitheliosa* were reported from neotropical areas of Latin America (Buyck 1988), as were six species from tropical areas of Africa (Buyck 1994; Buyck and Sharp 2007) and Madagascar (Heim 1938), and two species from arid areas of Australia (Lebel and Tonkin 2007, Hyde et al. 2016). Finally, six species native to Asia have been described, two from East Asia (Chiu 1945; Hongo 1968), and four from India (Das et al. 2005, 2017; Crous et al. 2016; Hyde et al. 2016).

Historically, taxonomic studies of *Russula* in Asia have been influenced by European or North American literature. That is why *Russula* species from Asia were often assigned names of morphologically similar counterparts from Europe or North America. However, recent molecular studies have revealed that many Asian *Russula* species are in fact not conspecific with European or North American species (Park et al. 2014; Lee et al. 2017; Paloi et al. 2018; Song et al. 2018). Although molecular data can provide additional information that may result in more robust phylogenies (Hebert et al. 2003; Savolainen et al. 2005; Hibbett et al. 2016; Adamčík et al. 2019), the use of this type of data may be hampered by misidentifications when there are limited reference databases or when low-resolution markers are used (Hofstetter et al. 2019).

The present study aims to: 1) clearly distinguish species of Russula subsection Amoeninae from different continents through a phylogenetic analysis using updated sequence data; and 2) revise the taxonomy of Korean *Amoeninae* based on materials obtained from recent collections from different habitats and areas of the Korean peninsula. Four *Amoeninae* species were reported in South Korea: *R. amoena*, *R. bella*, *R. mariae*, and *R. violeipes* (Park et al. 2013; Lee et al. 2015). *Russula amoena* and *R. violeipes* were described from Europe, *R. bella* from Japan, and *R. mariae* from North America. With the increase in available *Russula* sequence data, taxonomists can investigate more precisely the boundaries and distribution of Korean species. Therefore, the present study also aims to verify whether species are conspecific between continents using internal transcribed spacer (ITS) sequences from GenBank and generated for this study in a first analysis and, in a second analysis, using a concatenated dataset of other molecular markers including the second largest subunit of RNA polymerase II (*rpb2*), mitochondrial small subunit ribosomal DNA region (mtSSU), and the translation elongation factor 1-alpha (*tef1α*).

## Methods

### Sampling

A total of 15 collections from the Korean peninsula were included in this study. All Korean specimens were deposited in the Seoul National University Fungus Collection (SFC) and The Herbarium Conservation Center of the National Academy of Agricultural Science (HCCN). Because of a paucity of available sequence data from other continents, eight additional specimens from USA, four from Europe, and one from India were sequenced; all non-Asian samples are from the Herbarium of Plant Science and Biodiversity Centre of the Slovak Academy of Sciences (SAV) (Table 1 and Suppl. material 2: Table S2).

**Table 1.** Specimens used for the multi-locus analyses in this study. Sequences produced in this study are presented in boldface.

Taxon	Herbarium no.	Locality	GenBank accession no.		
			*rpb2*	mtSSU	*tef1*α
Outgroup					
R. aff. delica	1119/BB 12.086	Italy	KU237879	KU237442	KU238020
*R. chloroides*	572/BB 07.209	Slovakia	KU237845	KU237407	KU237990
*R. herrerae*	239/BB 06.532	Mexico	KU237772	KU237330	KU237915
Other subgenera					
R. aff. griseobrunnea	741/BB 09.344	New Caledonia	KU237877	KU237440	KU238018
*R. cfr. liberiensis*	91/BB 06.184	Madagascar	KU237760	KU237318	KU237905
*R. compacta*	228/B 06.295	USA	KU237766	KU237324	–
*R. edulis*	579/BB 08.167	Madagascar	KU237850	KU237412	KU237993
*Russula* sp.	569/BB 06.066	Madagascar	KU237842	KU237404	KU237987
*Russula* sp.	570/BB 08.178	Madagascar	KU237843	KU237405	KU237988
Closely related groups in subg. Heterophyllidiae					
R. aff. crustosa	31/BB 06.616	Canada	KU237747	KU237305	KU237896
R. aff. madagassensis	93/BB 06.255	Madagascar	KU237761	KU237319	KU237906
R. aff. virescens	721/BB 09.021	New Caledonia	KU237868	KU237430	KU238009
*R. amoenolens*	577/ BB 08.675	Italy	KU237410	KU237848	–
*R. amoenolens cfr. annulata*	75/BB 06.048	Madagascar	KU237756	KU237314	KU237902
*R. amoenolens cfr. illota*	36/ BB 06.380	Mexico	KU237750	KU237308	KU237898
*R. amoenolens cfr. pseudocarmesina*	6/BB 06.030	Madagascar	KU237739	KU237296	–
*R. amoenolens cfr. roseoalba*	82/BB 06.105	Madagascar	KU237758	KU237316	–
*R. amoenolens cfr. vesca*	45/BB 06.525	Mexico	KU237751	KU237309	KU237899
R. flavobrunnea var. violaceotincta	71/ BB 06.050	Madagascar	KU237754	KU237312	KU237901
*R. grisea*	449/BB 07.184	Slovakia	KU237795	KU237355	KU237939
*R. ionochlora*	448/BB 07.338	Slovakia	KU237794	KU237354	KU237938
*R. langei*	450/ BB 07.792	France	KU237796	KU237356	KU237940
*R. madagassensis*	21/BB 06.146	Madagascar	KU237742	KU237300	KU237891
*R. maguanensis*	XHW4765	China	MH939989	–	MH939983
*R. medullata*	555/BB 07.252	Slovakia	KU237832	KU237392	KU237976
*R. mustelina*	1176/SA 09.88	Slovakia	KU237881	KU237444	KU238022
*R. oleifera*	254/BB 98.024	Tanzania	KU237776	KU237334	KU237919
*R. ornaticeps*	46/BB 06.530	Mexico	KU237752	KU237310	–
*R. prolifica*	18/BB 06.161	Madagascar	KU237741	KU237299	KU237890
*R. pulverulenta*	578/ BB 05.160	USA	KU237849	KU237411	–
*Russula* sp.	545/BB 08.061	Madagascar	KU237823	KU237383	KU237967
*R. substriata*	XHW4785	China	MH939994	–	MH939988
Subsect. Amoeninae					
**R. aff. mariae**	**SAV F–4484**	**USA, New York State**	–	**MT417192**	–
**R. aff. mariae**	**SAV F–4493**	**USA, New York State**	–	**MT417193**	**MT417213**
**R. aff. mariae**	**SAV F–4564**	**USA, New York State**	–	**MT417194**	**MT417214**
***R. alachuana***	**SAV 1252**	**USA, Florida**	**MT417198**	**MT417186**	**MT417204**
***R. alachuana***	**SAV F–20108**	**USA, Florida**	**MT417199**	**MT417187**	**MT417206**
***R. amoena***	**SAV F–1352**	**Slovakia**	**MT417200**	**MT417185**	–
***R. amoena***	**SAV F–3147**	**Slovakia**	**MT417202**	**MT417190**	**MT417211**
**R. cf. amoenicolor**	**SAV F–20302**	**Greece**	**MT417196**	**MT417188**	**MT417209**
**R. cf. amoenicolor**	**SAV F–20324**	**Greece**	**MT417197**	**MT417189**	**MT417210**
***R. bella***	**SFC20120722–03**	**South Korea**	**MT199642**	**MT196930**	–
***R. bella***	**SFC20170819–05**	**South Korea**	**MT199643**	**MT196931**	**MT199655**
***R. bella***	**SFC20170819–10**	**South Korea**	**MT199644**	**MT196932**	**MT199656**
***R. bella***	**HCCN16818**	**South Korea**	KF361734	**MT196933**	–
***R. bella***	**HCCN15410**	**South Korea**	–	**MT196934**	–
***R. bella***	**HCCN21655**	**South Korea**	KF361736	**MT196935**	**MT199657**
***R. bella***	**SFC20170731–02**	**South Korea**	**MT199645**	**MT196936**	**MT199658**
*R. mariae*	546/BB 07.038	USA	KU237824	KU237384	KU237968
***R. orientipurpurea* sp. nov.**	**HCCN19111**	**South Korea**	KF361712	**MT196923**	**MT199648**
***R. orientipurpurea* sp. nov.**	**HCCN18725**	**South Korea**	KF361710	**MT196924**	**MT199649**
***R. orientipurpurea* sp. nov.**	**HCCN21685**	**South Korea**	KF361714	**MT196925**	**MT199650**
***R. orientipurpurea* sp. nov.**	**SFC20170819–08**	**South Korea**	**MT199638**	**MT196926**	**MT199651**
***R. orientipurpurea* sp. nov.**	**SFC20170725–37**	**South Korea**	**MT199639**	**MT196927**	**MT199652**
***R. orientipurpurea* sp. nov.**	**SFC20170821–22b**	**South Korea**	**MT199640**	**MT196928**	**MT199653**
***R. orientipurpurea* sp. nov.**	**SFC20170726–47**	**South Korea**	**MT199641**	**MT196929**	**MT199654**
***R. pseudoamoenicolor***		**India**	**MT199646**	**MT196937**	**MT199659**
*R. violeipes*	542/BB 07.273	Slovakia	KU237820	KU237380	KU237964
***Russula* sp.**	**SFC20160726–13**	**South Korea**	**MT199647**	**MT196938**	**MT199660**
***Russula* sp.**	**SAV F–20134**	**USA, Florida**	–	–	**MT417205**
***Russula* sp.**	**SAV F–20117**	**USA, Florida**	**MT417195**	–	**MT417208**
***Russula* sp.**	**SAV F–4063**	**USA, Tennessee**	**MT417203**	**MT417191**	**MT417212**

### Morphological study

Macromorphological characters were described from fresh specimens using the terminology of Vellinga (1988). The color standard codes in Kornerup and Wanscher (1978) were followed for describing the colour of the basidiomes. All microscopic characters were measured from dried herbarium samples using an Eclipse 80i light microscope (Nikon, Japan) with immersion lenses at the magnification of 1000× and using the software NIS ELEMENT BR v3.2 (Nikon, Japan). The description templates and terminology of Adamčík et al. (2019) were used for the observations of microscopic structures. The exception is that the sterile elements in hymenium have no distinct heteromorphous contents unlike hymenial cystidia of majority of *Russula* members, i.e. gloeocystidia. Because it is not certain if they correspond to “true gloeocystidia”, we refer to them as hymenial cystidia when observed on lamellae sides and marginal cells in case of lamellar edges. Spore ornamentation was observed using a light microscope, and a scanning electron microscope (SEM, SUPRA 55VP, Carl Zeiss, Germany) at 5,000× and 10,000× magnification. For each collection, statistics of the measurements of microscopic characters were based on 20 measurements per character. Spore measurements excluded ornamentation. We followed the protocols of chemical tests for micro-morphological observation in Adamčik et al. (2019). Statistics of microscopic characters are expressed as the mean ± standard deviation with extreme values in parenthesis. The mean values are indicated by underline. When multiple samples were available, individual measurements of all microscopic characters of a species were obtained from at least three samples and diagnostic characters of species were further used to compare with the remaining samples.

### Molecular studies

DNA was extracted from fresh or dried basidiomes using a modified CTAB extraction method (Roger and Bendich 1994). Four molecular markers were used for species-level identification and to infer evolutionary relationships among species. The following primer pairs were used in the amplifications: NSI1 and NLB4 for the ITS region (Martin and Rygiewicz 2005), bRBP2-6F1 and RPB2-7R for the partial *rpb2* locus (Matheny et al. 2007), MS1 and MS2 for part of the mtSSU region (White et al. 1990), and EF1-983F and EF1-2218R for the partial *tef1α* locus (Matheny et al. 2007). The PCR conditions were: initial denaturation of 5 min at 95 °C, 35 cycles that varied for each marker (60 s at 95 °C, 40 s at 50 °C, and 60 s at 72 °C for ITS; 40 s at 95 °C, 40 s at 58 °C, and 60 s at 72 °C for *rpb2*; 30 s at 94 °C, 30 s at 55 °C, and 60 s at 72 °C for mtSSU; 30 s at 94 °C, 30 s at 56 °C, and 60 s at 72 °C for *tef1α*), and final incubation of 7 min at 72 °C. All PCR products were checked on 1% agarose gel stained with EcoDye DNA staining solution (SolGent Co., Daejeon, South Korea) and purified with the Expin PCR purification kit (GeneAll Biotechnology, Seoul, South Korea) following the manufacturer’s instructions. DNA sequencing was conducted using an ABI3730 automated DNA Sequencer by Macrogen (Seoul, South Korea). The obtained sequences were checked and manually edited using the software FINCHTV v1.4 (Geospiza, Inc.), and then assembled manually using MEGA 7 (Kumar et al. 2016).

### Phylogenetic analysis

For species delimitation of the Korean samples, ITS sequences of R. subsect. Amoeninae were downloaded from GenBank and aligned with the newly generated ITS sequences using MAFFT v7, with the E-INS-I strategy (Katoh and Standley 2013). *Russula grisea* and *R. virescens* were used as outgroup based on the results of previous studies (Buyck et al. 2008; Park et al. 2013). Maximum Likelihood (ML) analyses were conducted using RAxML 8.2.10 (Stamatakis 2014) and the GTR + G model with 1000 rapid bootstrap replicates. For *rpb2*, mtSSU, and *tef1α* regions, sequences of each locus were separately aligned and analyzed after introns were excluded. Seven partitions were assigned; mtSSU, *rpb2*pos1, *rpb2*pos2, *rpb2*pos3, *tef1α*pos1, *tef1α*pos2, and *tef1α*pos3. Substitution models of all partitions were tested using ModelTest-NG (Darriba et al. 2020). The best substitution models for the different loci under BIC were GTR+I+G for mtSSU, K80+I+G for *rpb2* partitions, and TrN+I+G for *tef1α* partitions. Bayesian inference (BI) analysis was performed with MRBAYES v. 3.2.6 (Ronquist and Huelsenbeck 2003), with four independent runs of four chains each. The TrN substitution model for *tef1α* was replaced by the GTR model for this analysis. The analysis was run for 20 million generations, with sampling every 1,000^th^ generation. At the end of the run, the average standard deviation of split frequency of runs was 0.001412. The convergence and burn-in values of runs were then checked in Tracer 1.6 (Rambaut et al. 2014). We considered clades with the bootstrap values and posterior probabilities exceeding 70% and 0.95 as well-supported. The ITS dataset and the combined dataset (*rpb2*-mtSSU-*tef1α*) are available in TreeBase (http://treebase.org/treebaseweb/; submission ID 26896 and 22640, respectively). All phylogenetic analyses were executed on the CIPRES Science Gateway (Miller et al. 2010). Three species of R. subg. Malodorae were chosen as outgroup.

## Results

### Phylogenetic analysis

**Figure 1.**
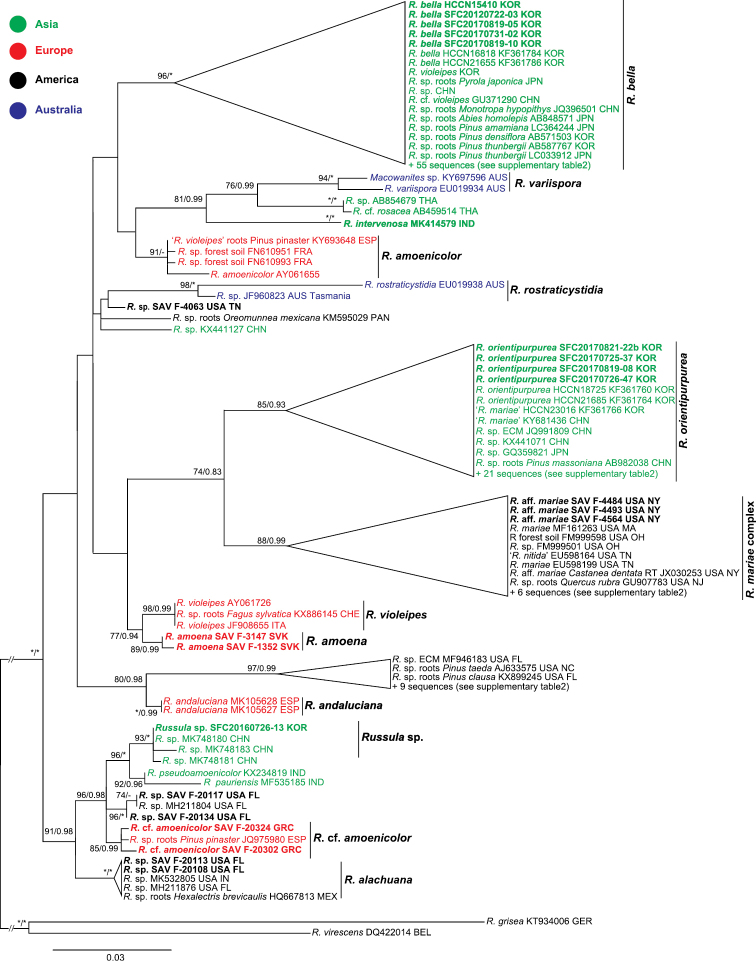
Maximum Likelihood (ML) tree based on internal transcribed spacer (ITS) sequences of most Russula subsect. Amoeninae and closely related species. Species in boldface are described in this study. ML bootstrap values >70 and Bayesian Inference posterior probability >0.90 are shown. Stars indicate clades with 100 ML bootstrap values and 1.0 Bayesian posterior probabilities.

The ITS region was amplified and sequenced from 22 specimens for this study. A total of 152 ITS sequences belonging to *Amoeninae* were downloaded from GenBank and used in the analysis (Suppl. material 2: Table S2). The phylogenetic analysis of the ITS sequences indicated the existence of more than 21 *Russula* species-level clades: eight Asian species (two names undetermined), five European (one undetermined), five North American (three undetermined), two Australian, and one Central American (undetermined) (Fig. 1). However, none of the African or Malagasy species were included in this analysis as no ITS sequences were available. The Korean samples represented three phylogenetic species, and they were grouped with Asian samples and clearly separated from specimens of Australia, Europe, and North America.

**Figure 2.**
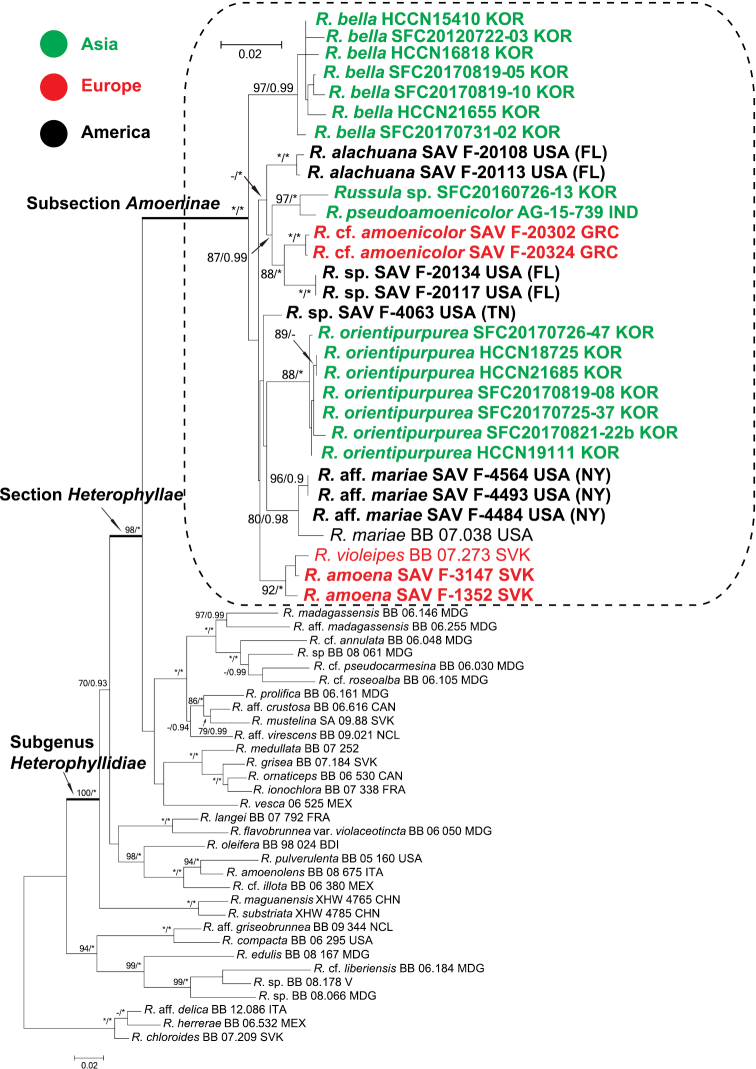
Maximum Likelihood (ML) tree based on the second largest subunit of RNA polymerase II (*rpb2*), mitochondrial small subunit ribosomal DNA region (mtSSU), and the translation elongation factor 1-alpha (*tef1α*) sequences of Russula subsect. Amoeninae species and representatives from closely related subgenera. Species in boldface are described in this study. ML bootstrap values >70 and Bayesian Inference posterior probability >0.90 are shown. Stars indicate clades with 100 ML bootstrap values and 1.0 Bayesian posterior probabilities.

A total of 72 ITS sequences were confirmed as *R. bella*: 5 from this study and 67 from GenBank. All of these sequences are from specimens in East Asia, i.e. from South Korea, China, and Japan. Of the ITS sequences in the *R. bella* clade, 31 were initially misidentified as the European *R. violeipes*, 35 ambiguously labelled as “*Russula* sp.”, and one labelled as Russula cf. violeipes. A total of 33 specimens for which ITS sequences were newly generated or retrieved from GenBank belonged to a new species clade, *R. orientipurpurea.* The twenty nine ITS sequences from GenBank originated from South Korea, China, and Japan. Of these, 22 were mislabelled as the North American *R. mariae* and seven were labelled as “*Russula* sp.” (Suppl. material 1: Table S1). The *R. orientipurpurea* clade was closely related to *R. mariae*, but they were clearly separated (Figs 1, 2). One specimen (SFC20160726-13) formed a unique clade with three Chinese specimens and we define it as an undetermined species. Two Indian species were positioned as a sister clade of the *Russula* sp. clade.

*Russula amoena*, *R. amoenicolor*, *R. andaluciana*, and *R. violeipes* were monophyletic (Fig. 1). The European Mediterranean samples have similar morphology with *R. amoenicolor*. However, phylogenetic analysis showed that they are likely not conspecific. Therefore, we named them as “R. cf. amoenicolor” in this study. The North American samples formed five clades. Of these, only two clades are labelled with species names (*R. mariae* and *R. alachuana*). The four Australian samples formed two well-supported clades that do not overlap with samples from other continents.

Sequences of three loci (*rpb2*, mtSSU, and *tef1α*) were obtained for 28 samples (Table 1) and combined with 102 sequences of 34 samples obtained from GenBank. Two short introns were detected only in *tef1α* and they were excluded in the phylogenetic analysis. The results of this multilocus phylogenetic analysis were similar to those of the ITS analysis. Subsection Amoeninae formed a well-supported monophyletic group (Fig. 2). Three species were found for South Korea, and the East Asian *Russula* species were clearly separated from European and North American species.

### Morphological analysis

**Figure 3.**
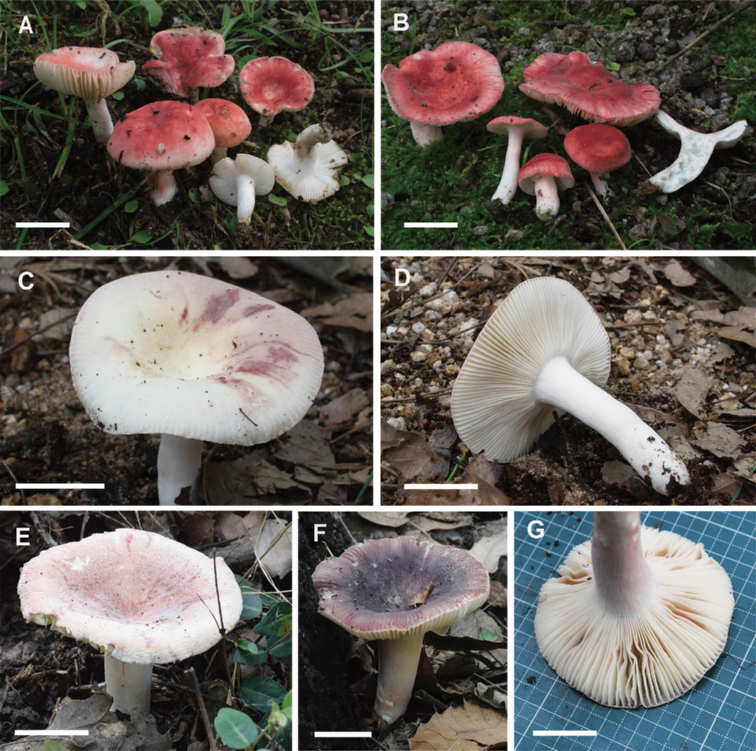
Basidiomes of Korean *Amoeninae* from this study. **A, B***Russula bella* (A-SFC20170802-03, B-SFC20170819-05) **C–E***R. orientipurpurea* (SFC20170819-08) **F, G***Russula* sp. (SFC20160726-13). Scale bars: 20 mm.

**Figure 4.**
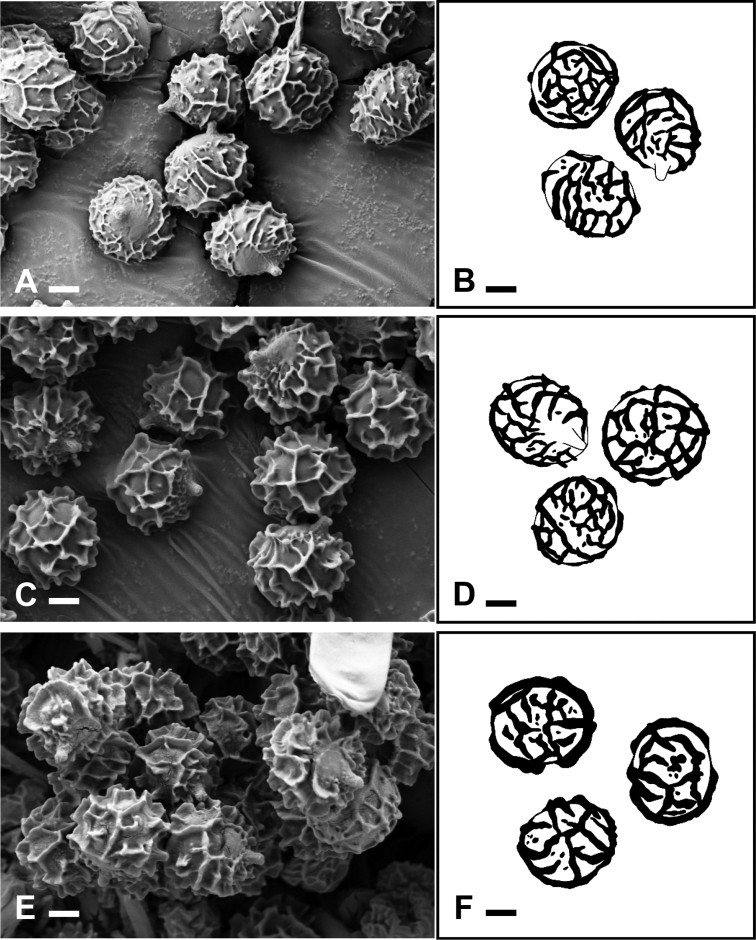
Basidiospores with scanning electron microscopy images (**A, C, E**) and drawing images (**B, D, F**) **A, B***Russula bella* (SFC20170819-05) **C, D***R. orientipurpurea*; (SFC20170821-22b) **E, F***Russula* sp. (SFC20160726-13). Scale bars: 2 µm.

**Figure 5.**
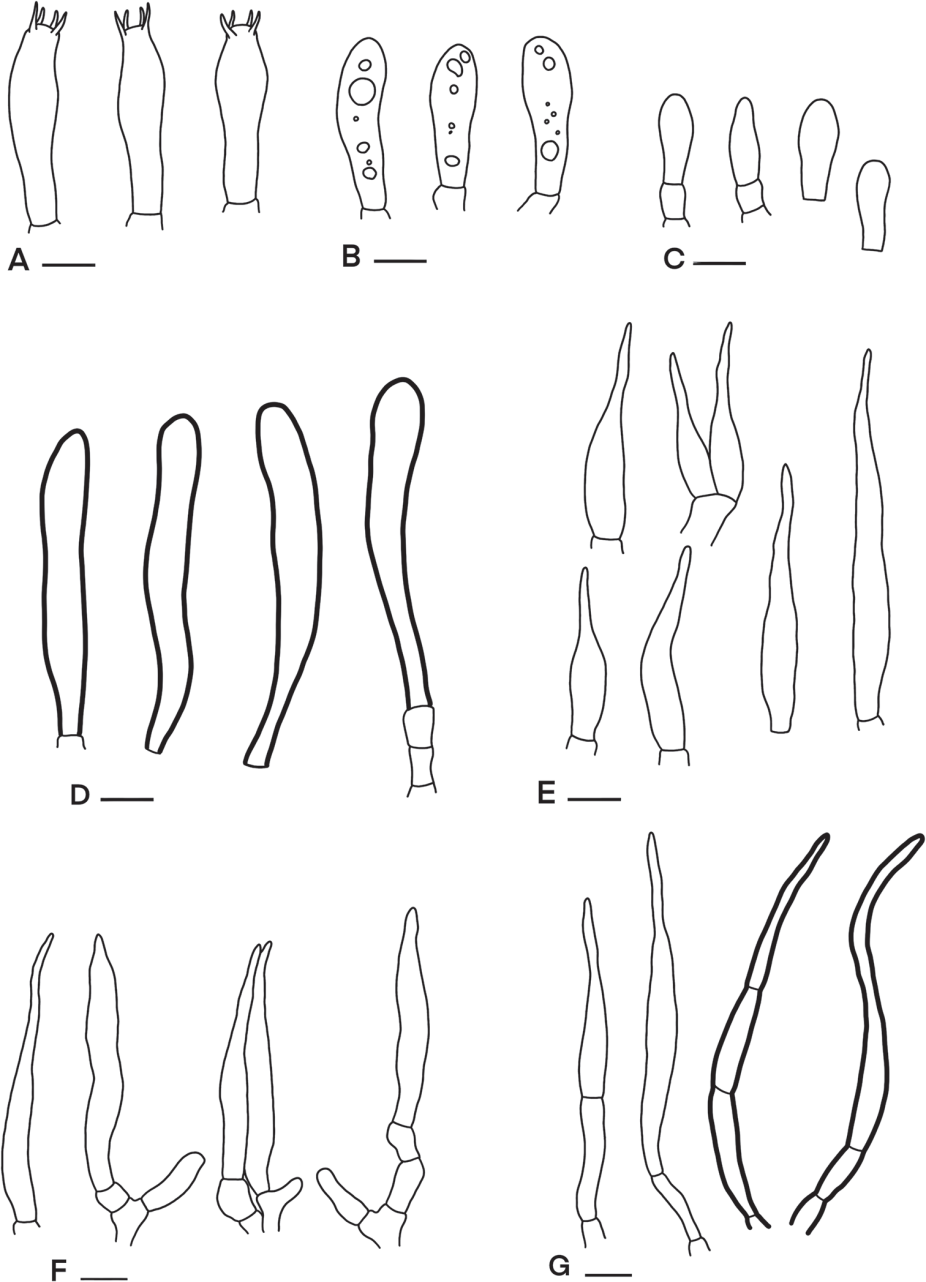
Microscopic features of *Russula bella* (SFC20170819-05) **A** basidia **B** basidiola **C** clavate marginal cells **D** hymenial cystidia on lamellae sides **E** subulate marginal cells **F** hyphal termination at pileus margin **G** hyphal termination at pileus centre. Scale bars: 10 µm.

**Figure 6.**
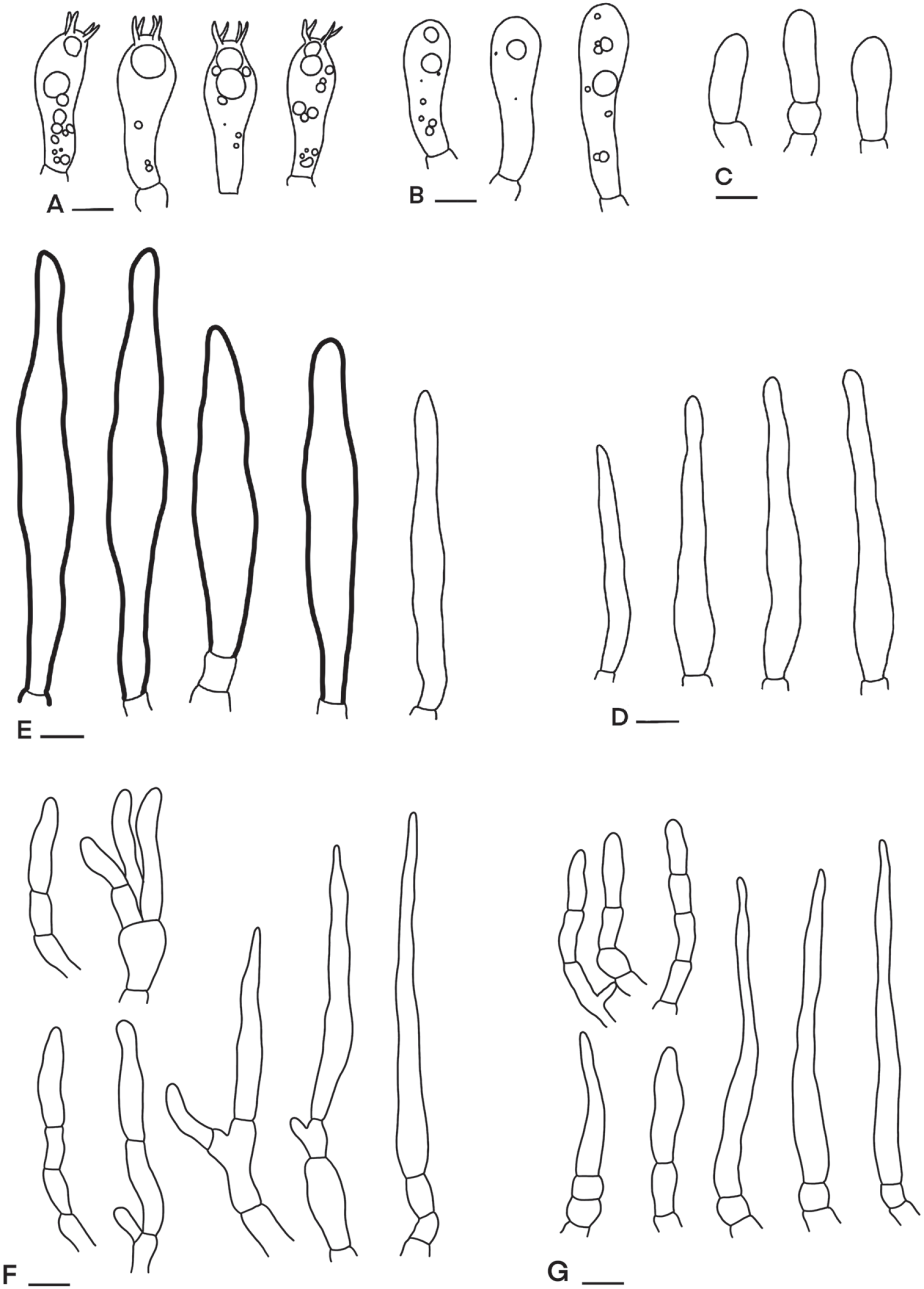
Microscopic features of *Russula orientipurpurea* (SFC20170725-37) **A** basidia **B** basidiola **C** clavate marginal cells **D** hymenial cystidia on lamellae sides **E** subulate marginal cells **F** hyphal termination at pileus margin **G** hyphal termination at pileus centre. Scale bars: 10 µm.

**Figure 7.**
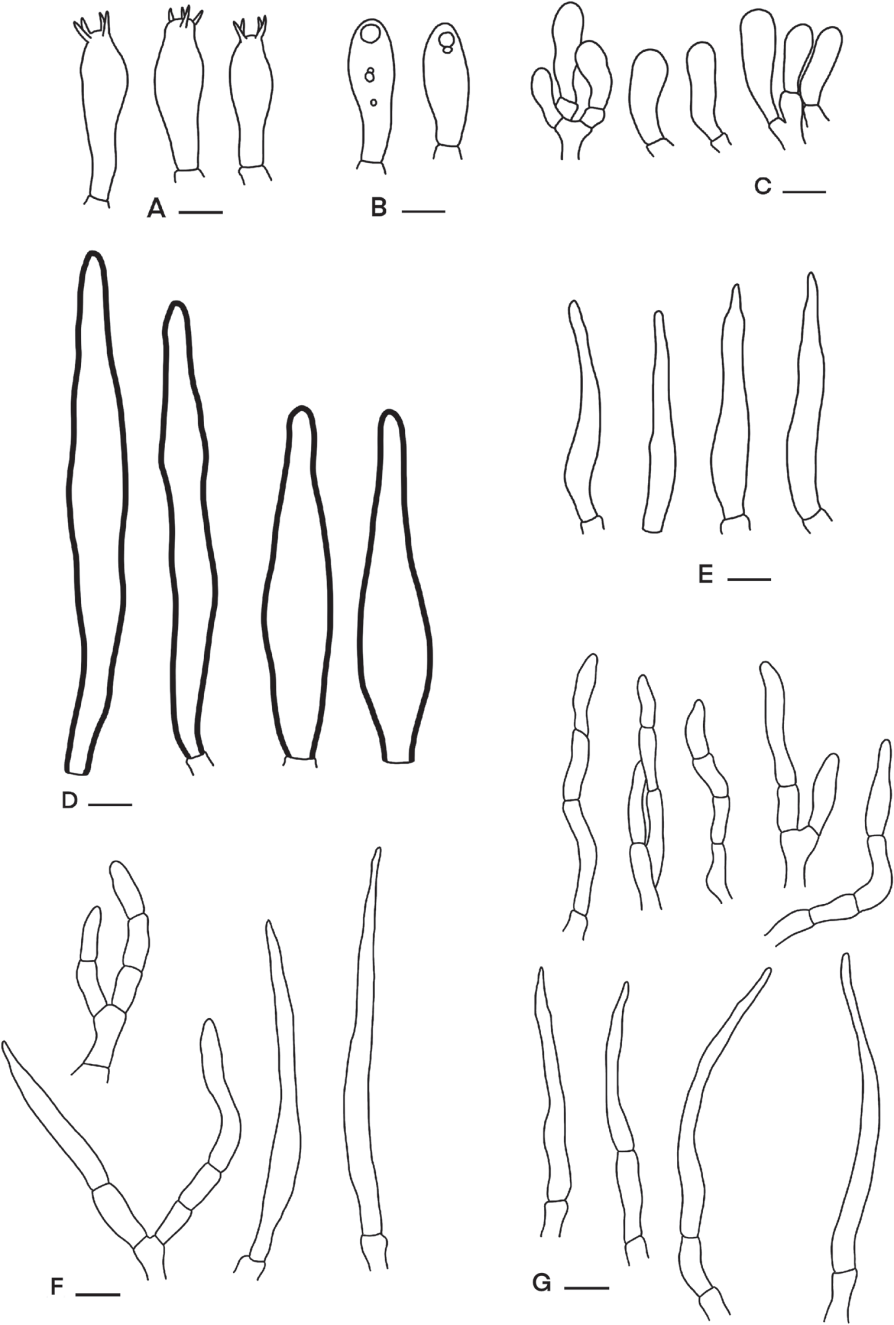
Microscopic features of *Russula* sp. (SFC20160726-13) **A** basidia **B** basidiola **C** clavate marginal cells **D** hymenial cystidia on lamellae sides **E** subulate marginal cells **F** hyphal termination at pileus margin **G** hyphal termination at pileus centre. Scale bars: 10 µm.

The three species found in South Korea have the typical morphological characters of subsection Amoeninae: pruinose dry pileus surface, mild taste, and stipe flushed with pink, red, or purple. In fact, they have almost completely white stipes, usually with only a faint pink color (Fig. 3). Microscopically, these species display moderately large spores with crested and subreticulate to reticulate ornamentation (Fig. 4), an absence of cystidia with well-defined contents reactant with sulfovanillin, and a pileipellis comprising mainly attenuated terminal cells of hyphae usually subtended by one or two shorter ellipsoid or subglobose cells. The pileipellis of all three species have dimorphous hyphal terminations, some with long subulate or lageniform terminal cells and others with shorter, cylindrical, or ellipsoid terminal cells (Figs 5–7). The three Korean species were easily distinguished in the field. The morphological features of the Korean *R. bella* are consistent with the original description of the species (Table 2). The other two Korean species, however, do not entirely agree with the description of any previously described *Russula* species.

**Table 2.** Morphological characteristics of Asian R. subsect. Amoeninae species.

		*R. bella* ^a^	***R. bella***	*R. intervenosa* ^b^	*R. mukteshwarica* ^c^	***R. orientipurpurea***	*R. pauriensis* ^d^	*R. pseudoamoenicolor* ^e^	*R. punicea* ^f^	***Russula* sp.**
Pileus size (mm)		20–45	20–50	26–49	65–130	52–60	53–63	50–100	25–60	60
Pileus colour	bright red	√	√	√				√		
	pink	√	√	√					√	
	grey					√	√			√
	brown									
	purple				√	√		√		
	violet				√		√	√		√
	green				√		√			
	bright yellow			√	√					
	cream or pale yellow					√	√			
Stipe colour	almost white		√			√			√	√
	partly pink	√	√	√	√			√		
	partly purple			√	√		√	√		
	partly violet						√			
Spore size	length (µm)	6.5–7.5	6.5–7.7	7–8	7.6–9.3	6.9–7.8	6–8	6–9.5	6.5–7	6.5–7
	width (µm)	5.5–6	5.3–6.0	6.5–7	7.3–8.2	6–6.9	5.5–7	5–8	5–6	5.6–6.2
	mean (length × width)		7.1 × 5.7	7.5 × 6.7		7.3 × 6.4	6.9–6.3	7.3–6.3		6.8 × 5.9
	Q value		1.17–1.33	1.07–1.19	1.00–1.15	1.09–1.19	1.00–1.17	1.03–1.33		1.10–1.19
Spore ornamentation	subreticulate	√	√	√	√	√	√	√	√	√
	reticulate					√			√	
	height (µm)		0.4–1.0	0.6–0.9	0.75	0.6–1	-2	-2		0.7–1.2
Cystidia on lamellae sides	length (µm)	44–55	52–75	29–34	80–110	74.5–101	55–135	90–117	45–60	66.5–91.5
	width (µm)	5.5–7	7.5–10.5	10–12.5	11–17	10.5–15	12–22	10–21	9–12	12.5–16.5
	cylindrical or clavate	√	√						√	
	subulate or lageniform									√
	fusiform			√	√	√	√	√	√	√
	obtuse		√			√	√	√	√	
	acute			√			√		√	√
Cystidia on lamellae edges	length (µm)	37–65	38.5–63	32–39	70–100	48–88	36–68	30–85		42.5–56.5
	width (µm)	5.5–7	5.5–7.5	5.5–7	11–17	5.5–10.5	8–15	7–10		5.5–7
	different from sides	√	√	√						
TC (margin)	length (µm)	44–80	47–76	39–47		55.5–89	9–64	11–65		60–85
	width (µm)	5.3–8	5–7	2.5–4.5	5–11	5–7	4–10	4–10	6–9	4.5–6
Short cells	number		1–2		2–4	0–2	2–4			1–2
	subterminal width (µm)						-12	-14		
TC centre	width (µm)		4.5–7.5			4.5–7.5				3–4

References: a-Hongo 1968, b-Crous et al. 2016, c-Das et al. 2005, d-Das et al. 2017, e-Hyde et al. 2016, f-Chiu 1945, Size unit of pileus is mm and the other microscopic characters are µm. Species in boldface are described in this study.

## Taxonomy

### Russula bella

Taxon classificationFungiRussulalesRussulaceae

Hongo, Memoirs of Shiga University 18:50 (1968)

01ADF8C7-693F-504F-91A2-3BAB8ABB498B

Figs 3A, B , 4A, B , 5

#### Diagnosis.

**Pileus** medium-sized, 20–50 mm diam., plano-convex to convex when young, applanate with depressed center to infundibuliform when mature, often lobate, margin with short striation, sometimes cracking in age; cuticle smooth, pruinose, viscid and shiny when wet, cuticle peeling approximately to half of the pileus radius or sometimes almost to the center, color variable, typically darker at the disk, greyish red (11D5), brownish violet (11D6) to bluish red (12A6-A8, 12B7), few with yellowish brown spots, margin pink (11A5) to greyish rose (11B5). **Lamellae** 2–3 mm deep, brittle, adnate, approximately 11–19 per cm near the pileus margin, moderately distant to crowded, yellowish white to pale cream; lamellulae absent; edge entire and concolorous. **Stipe** 17–34 × 5–9 mm, centrally attached or eccentric, cylindrical to slightly tapered towards the base, surface dry, longitudinally striate, whitish with a pinkish flush; solid when young, becoming hollow in age. **Context** 1–3 mm thick near the stipe, white, unchanging after cutting, turning greenish with FeSO_4_, turning quickly yellow with KOH, pale violet with PDAB; taste mild; odor slightly fruity. **Spore print** pale cream to white.

**Basidiospores** (n = 60, 3, 3) (6–)6.5–7.1–7.7(–8.8) × (5–)5.3–5.7–6(–6.6) µm, broadly ellipsoid, Q = (1.11–)1.17–1.25–1.33(–1.49), ornamentation thin, 0.4–1 µm high ridges forming an incomplete reticulum (2–7 in a 3 µm circle) with some dispersed isolated warts (0–2 warts in a 3 µm circle), suprahilar spot not amyloid, smooth. **Basidia** (25.1–)30–35.3–40.5(–48) × (7–)8.5–9.4–10.5(–11.5) µm, 4-spored, narrowly clavate, with guttate or granular contents. **Basidiola** (27.3–)28.4–32–35.5(–41.7) × (8.1–)8.7–9.4–10.3(–10.6) µm, narrowly clavate, with guttate or granular contents. **Hymenial cystidia on lamellae sides** inconspicuous, widely dispersed, up to 100 per mm^2^, (37.5–)52–63.5–75(–90) × (5.5–)7.5–9–10.5(–11.5) µm, clavate to subcylindrical, originating from subhymenium, apically obtuse, thick-walled with walls up to 0.8 µm; contents optically empty, negative in sulfovanillin. **Lamellar edge** with dispersed basidia, true gloeocystidia (with differentiated contents) absent; marginal cells very abundant, resembling terminal cells in the pileus, typically narrowly lageniform or subulate (24–)38.5–50.8–63(–83) × (4–)5.5–6.8–7.5(–9.5) µm; shorter clavate to subcylindrical with obtuse apex present, (9–)12.5–15.7–18.5(–23.5) × (3.5–)4.5–5.7–7(–7.5) µm. **Pileipellis** orthochromatic in Cresyl blue, trichoderm, sharply delimited from the underlying context, 280‒400 µm thick, with a well-defined, gelatinized, 150‒200 µm thick suprapellis of ascending or erect hyphal terminations forming a trichoderm, subpellis 130–200 µm thick, dense, horizontally oriented, sldender and gelatinized hyphae; acid-resistant incrustrations absent. Hyphal terminations near the pileus margin unbranched, often slightly flexuous, either long and attenuated or subcylindrical and obtuse, the attenuated ones more abundant, with terminal cells (20.5–)47–61.4–76(–85) × (3.5–)5–5.9–7(–8) µm, subulate or narrowly lageniform, rarely fusiform, apically acute to subacute, thin-walled, usually followed by 1–2 shorter and often more inflated cells before the branching; subcylindrical ones shorter, with terminal cells (7.5–)33.5–51.2–69(–107.5) × (2–)3.5–4.7–6(–7) µm, frequently originate from branched cells, sometimes with one shorter unbranched subterminal cell. Hyphal termination near the pileus center also dimorphous, the attenuated ones with terminal cells (10.5–)18.5–50.5–82.5(–104.5) × (3.5–)4.5–6.0–7.5(–10) µm, mainly subulate, occasionally narrowly fusiform, apically acute and sometimes acute-pointed, often with thickened walls (up to 0.8 µm), shorter cylindrical hyphae with terminal cells (9–)12.5–18.6–24.5(–32) × (2.5–)3–4–5(–6) µm; followed by 1–3 unbranched shorter cells, subterminal cells not usually not distinctly wider. **Pileocystidia** absent. Cystidioid or oleipherous hyphae in subpellis or context absent.

#### Ecology.

gregarious to scattered on soil in mixed forest with *Quercus aliena*, *Pinus densiflora*, and *Populus* sp.

#### Studied materials.

South Korea. Jeollanam-do, Haenam-gun, Mt. Duryun, 614 m elev., 34°27'24"N, 126°37'07"E, Yang Seop Kim, 5 September 1985, HCCN1457A (HCCN!); Chungcheongbuk-do, Danyang-gun, Mt. Sobaek, 790 m elev., 36°57'29"N, 128°26'35"E, Soon Ja Seok, 13 July 2007, HCCN15410 (HCCN!); Gyeonggi-do, Suwon-si, Seonggyungwan University, 58 m elev., 37°17'42"N, 126°58'22"E, Soon Ja Seok, 1 August 2008, HCCN16735 (HCCN!); Gangwon-do, Wonju-si, 275 m elev., 37°19'59"N, 127°54'35"E, Soon Ja Seok, 6 August 2008, HCCN16818 (HCCN!); Gyeonggi-do, Suwon-si, Seonggyungwan University, 48 m elev., 37°17'28"N, 126°58'24"E, Hye Yeong Choi, 5 August 2011, HCCN21655 (HCCN!); Daejeon-si, Yuseong-gu, 105 m elev., 36°23'48"N, 127°20'13"E, Myung Soo Park, 31 July 2012, SFC20120731-02 (SFC!); Gyeongsangbuk-do, Ulleung-gun, Mt. Seonginbong, 420 m elev., 37°30'50"N, 130°52'10"E, Seung-Yoon Oh, Won Ju Kim, Young Woon Lim, 14 August 2012, SFC20120814-23 (SFC!); Chungcheongnam-do, Seosan-si, Yonghyeon Natural Recreation Forest, 151 m elev., 36°45'53"N, 126°36'10"E, Young Ju Min, Won Ju Kim, Hyun Lee, 10 October 2012, SFC20121010-06 (SFC!); Seoul, Gwanak-gu, Seoul National University, 103 m elev., 37°27'26"N, 126°56'59"E, Komsit Wisitrassameewong, 31 July 2017, SFC20170731-02 (SFC!); ibid., 19 August 2017, SFC20170819-05 (SFC!).

#### Comments.

*Russula bella* is morphological similar to *R. pseudoamoenicolor*, *R. punicea*, and *R. violeipes*. *Russula pseudoamoenicolor* has a more vividly colored pileus and a larger basidiome and hymenial cystidia than those of *R. bella* (Hyde et al. 2016). *Russula punicea* differs from *R. bella* in the shape of hymenial cystidia; the former has acute cystidia, whereas the latter has obtuse cystidia (Chiu 1945; Hongo 1968). *Russula violeipes* differs from *R. bella* in the yellowish to greenish smeared violet color of the pileus (Kränzlin 2005). Moreover, *R violeipes* has larger basidia (45–65 × 11–14 µm) and pleurocystidia (80–115 × 12–15 µm) than those of *R. bella* (Kränzlin 2005).

### Russula orientipurpurea

Taxon classificationFungiRussulalesRussulaceae

Wisitr., H. Lee & Y.W. Lim sp. nov.

47144CFE-DB12-566F-978B-B64361E338E2

MycoBank No: 835272

Figs 3C–E , 4C, D , 6

#### Material examined.

***Holotype***. South Korea. Jullanam-do, Yeosu-si, Dolsando islands, 202 m elev., 34°35'24"N, 127°47'57"E, Komsit Wisitrassameewong, Jae Young Park, 25 July 2017, SFC20170725-37 (Holotype, SFC!).

#### Etymology.

‘orientipurpurea’ refers to the origin of the species, East Asia, and its typical purple color of pileus.

#### Diagnosis.

Pileus surface with pale cream with flushed pale purple to purple stains; spores with almost complete to complete reticulum; subfusiform to fusiform hymenial cystidia.

**Pileus** medium-sized, 52–60 mm diam., plano-convex to applanate with the deeply depressed center, margin inconspicuously striate up to 2 mm, acute, even; surface smooth, pruinose, slightly waxy, matt, slightly viscid when wet, cuticle peeling 1/2 to 3/4 of the pileus radius, color pale cream to cream, with darker shade of cream towards the center, typically flushed with pale or darker purple stains, sometimes with radial stripes of greyish ruby (12E5). **Lamellae** 4–5 mm deep, adnate, moderately distant, approximately 11–18 per cm near the pileus margin, white to pale yellow (3A3), furcations sometimes present near the stipe, lamellulae occasional, edge even. **Stipe** 40–50 × 11–13 mm, centrally attached, cylindrical, surface smooth, longitudinally striate, color white and sometimes with a greyish red (11D4-D5) flush; hollow. **Context** 2–3 mm thick at half of the pileus radius, white, rather firm but fragile in stipe when mature, turning slowly greenish with FeSO_4_ and pale orange to orange white with KOH; taste mild; odor slightly fruity. **Spore print** cream white to white.

**Basidiospores** (n = 80, 4, 4) (6.3–)6.9–7.3–7.8(–8.6) × (5.4–)6–6.4–6.9(–7.6) µm, subglobose to broadly ellipsoid, Q = (1.06–)1.09–1.14–1.19(–1.28), ornamentation of thin to moderately thick, 0.6–1.4 µm high ridges forming an incomplete or complete reticulum (3–7 in a 3 µm circle), in a 3 µm circle isolated warts rare (0–1 in a 3 µm circle), suprahilar spot not amyloid, small, surrounded by fine and less prominent reticulation. **Basidia** (28–)32–36.2–40(–45) × (7.5–)9.5–10.9–12.5(–14.5) µm, 4-spored, clavate. **Basidiola** (27.8–)33.4–37.7–42.1(–43.4) × (9–)9.7–10.5–11.4(–12.2) µm, narrowly clavate, with guttate or granular contents. **Hymenial cystidia on lamellae sides** widely dispersed to dispersed, 200–700 per mm^2^, (64–)74.5–87.9–101(–131) × (8.5–)10.5–12.8–15(–18.5) µm, mostly fusiform or narrowly fusiform, occasionally clavate, originating from subhymenium, emergent or not beyond basidia, apically constricted but obtuse, usually with thickened walls (up to 0.8 µm), contents optically empty, negative in sulfovanillin. **Lamellar edge** with dispersed basidia, true gloeocystidia (with differentiated contents) absent; marginal cells very abundant, resembling terminal cells in the pileus, usually narrowly lagenifom or subfusiform, apically narrowed but obtuse (28.5–)48–67.8–88(–121) × (3–)5.5–8.2–11(–14.5) µm; shorter narrowly clavate to clavate, (10.5–)13.5–18–22.5(–27.5) × (3.5–)5–6.5–8(–10.5) µm. **Pileipellis** orthochromatic in Cresyl blue, sharply delimited from the underlying context, 170–240 µm thick, with a well-defined, gelatinized, 30–60 µm thick suprapellis of ascending or erect hyphal terminations forming a trichoderm, subpellis 180–250 µm thick, dense, horizontally oriented, gelatinized and branched cylindrical to inflated hyphae; acid-resistant incrustrations absent. Hyphal terminations near the pileus margin unbranched, apically often flexuous, either long and attenuated or subcylindrical, short and obtuse, the attenuated ones (39.0–)55.5–72.3–89.0(–112.0) × (2.5–)5.0–6.1–7.2(–8.5) µm, subulate or narrowly fusiform, sometimes slightly moniliform, apically subacute, thin-walled, subterminal cells shorter, cylindrical ones with terminal cells (10–)16.5–25.3–34(–57) × (3–)4–5.2–6(–7.5) µm, apically sometimes slightly constricted, apically obtuse; followed by 0–2 unbranched short, equally wide cells, sometimes originate from branched cells. Hyphal termination near the pileus center also dimorphous, the attenuated ones prevailing with terminal cells (10.5–)18.5–50.5–82.5(–104.5) × (3.5–)4.5–6.0–7.5(–10) µm, subulate, thin-walled, apically subacute, cylindrical ones with terminal cells (13.5–)19–42.9–66.5(–85.5) × (3.5–)4.5–5.6–6.5(–9.0) µm. **Pileocystidia** absent. Cystidioid or oleipherous hyphae in subpellis or context absent.

#### Ecology.

solitary to scattered on soil in mixed forest with *Quercus* and *Pinus* trees.

#### Studied materials.

South Korea. Chungcheongnam-do, Gongju-si, Mt. Museong, 341 m elev., 36°35'52"N, 127°01'59"E, Hyun Lee, Seung-Yoon Oh, 26 July 2012, SFC20120726-37 (Paratype SFC!); Incheon-si, Gangwha-gun, Mt. Goryeo, 228 m elev., 37°44'54"N, 126°26'01"E, Young Woon Lim, 4 August 2012, SFC20120804-09 (Paratype, SFC!); Seoul, Gwanak-gu, Mt. Gwanak, 154 m elev., 37°27'06"N, 126°56'34"E, Hyun Lee, Won Ju Kim, 25 August 2012, SFC20120825-02 (Paratype SFC!); ibid, 202 m elev., 37°27'34"N, 126°56'19"E, Hyun Lee, Myung Soo Park, 31 August 2012, SFC20120831-04 (Paratype SFC!); ibid, 238 m elev., 37°26'53"N, 126°54'11"E, Hyun Lee, Komsit Wisitrassameewong, 19 August 2017, SFC20170819-08 (Paratype SFC!); Gyeongsangnam-do, Hapcheon-gun, Mt. Gaya, 631 m elev., 35°47'59"N, 128°05'46"E, Jae Young Park, Komsit Wisitrassameewong, Ki Hyeong Park, 26 July 2017, SFC20170726-47 (Paratype SFC!); Gyeongsangbuk-do, Ulleung-gun, Nari basin, 395 m elev., 37°31'03"N, 130°52'11"E, Jae Young Park, Nam Kyu Kim, 21 August 2017, SFC20170821-22b (Paratype SFC!).

#### Comments.

*Russula orientipurpurea* is common in mixed forests in South Korea. This species was misidentified as the North American *R. mariae* (Park et al. 2013). The spores in *R. orientipurpurea* have more complete ridge connections (3–7 lines in the 3 µm circle) and a smaller number of warts (0–1 warts in the 3 µm circle) than those in *R. mariae* (lines 1–4, warts 4–6). *Russula orientipurpurea* is morphologically similar to *R. intervenosa*. However, the dark red pileus centere, broader hymenial cystidia (up to 18 µm), and the thicker pileipellis (250–600 µm) of *R. intervenosa* (Crous et al. 2016) distinguishes this species from *R. orientipurpurea* (see Table 2).

### Russula

Taxon classificationFungiRussulalesRussulaceae

sp.

9701A921-8289-5DF9-8505-D2CE73E4F70C

Figs 3F, G , 4E, F , 7

#### Diagnosis.

**Pileus** medium-sized, 60 mm diam., applanate with deeply depressed center, margin distinctly striate, crenulate, radially cracking; cuticle dry, viscid when moist, pruinose, peeling to 1/2 of the pileus radius, color greyish violet (18E5-E6), with dark violet (18F4-F6) patches, towards margin violet grey (18D2) to dull violet (18D3). **Lamellae** 3 mm deep, adnate, dense, pale cream; lamellulae rare, forked near the stipe; edge smooth and concolorous. **Stipe** 55 × 8–10 mm, centrally attached, tapering downwards base, surface dry, longitudinally-striated, white and flushed with purple. **Context** 2 mm thick at half of the pileus radius, white, unchanging; taste and odor not recorded. **Spore print** cream white.

**Basidiospores** (n = 20, 1, 1) (6.4–)6.5–6.8–7(–7.2) × (5.5–)5.6–5.9–6.2(–6.5) µm, Q = (1.08–)1.1–1.14–1.19(–1.24), subglobose to broadly ellipsoid, ornamentation of thin to moderately thick, 0.8–1.4 µm high ridges forming an incomplete reticulum (2–6 in a 3 µm circle), isolated warts rare (0–2 in a 3 µm circle), suprahilar spot not amyloid, small. **Basidia** (32–)34–36.2–38.5(–40) × (9–)9.5–10.5–11(–11.5) µm, 4-spored, clavate. **Basidiola** (24–)25.8–31–35(–42.5) × (8.3–)8.9–9.9–10.8(–11.4) µm, narrowly clavate, with guttate or granular contents. **Hymenial cystidia on lamellae sides** widely dispersed to dispersed, 100–500 per mm^2^, (63)66.5–79–91.5(–109) × (10.5–)12.5–14.5–16.5(–18) µm, narrowly fusiform or lageniform, originating from subhymenium and emergent beyond basidia, apically acute or obtuse but narrowed, thick-walled (walls up to 0.9 µm), contents optically empty, negative in sulfovanillin. **Lamellar edge** with dispersed basidia, true gloeocystidia (with differentiated contents) absent; marginal cells very abundant, resembling terminal cells in the pileus, narrowly lageniform or subulate (36.5–)42.5–49.6–56.5(–70.1) × (5–)5.5–6.3–7(–7.5) µm, narrowly lageniform or subulate, apically acute; narrowly clavate to clavate with optuse apex, (11.5–)14.5–17.8–21.2(–23.2) × (4–)5.2–6.2–7(–7.5) µm. **Pileipellis** orthochromatic in Cresyl blue, sharply delimited from the underlying context, 250–400 µm thick, with gelatinous matter, cystidoid hyphae prevailing, shorter cylindrical terminal hyphae present, parallel or repent scattered, pileocystidia absent, with a well-defined, gelatinized, 70–120 µm thick suprapellis of ascending or erect hyphal terminations forming a trichoderm, subpellis 230–350 µm thick, dense, horizontally oriented, gelatinized and branched cylindrical hyphae; acid-resistant incrustrations absent. Hyphal terminations near the pileus margin unbranched, usually only slightly flexuous, either long and attenuated or subcylindrical, short and obtuse; the attenuated ones more frequent, with terminal cells (45.0–)60.0–72.6–85.0(–96.5) × (3.5–)4.5–5.1–6.0(–6.5) µm, subulate, apically acute, thin-walled, shorter ones with terminal cells (18–)20.5–28.7–37.0(–48.0) × (3.0–)3.5–4.4–5.0(–6.0) µm, cylindrical or subcylindrical, apically obtuse but often constricted, thin walled; followed by (0–)1–2(–3) unbranched shorter and equally wide cells. Hyphal termination near the pileus center similar but shorter and narrower, attenuated longer ones with terminal cells (38.5–)43.5–53.4–63.0(–73.0) × (2.5–)3–3.5–4.0(–4.5) µm; shorter cylindrical ones with terminal cells (11.5–)13.5–16.4–19.0(–22.0) × (3.0–)3.5–4.1–4.5(–5.5) µm. **Pileocystidia** absent. Cystidioid or oleipherous hyphae in subpellis or context absent.

#### Ecology.

solitary on soil in deciduous forest, near *Quercus mongolica*.

#### Material examined.

South Korea. Incheon-si, Ongjin-gun, Jangbongdo islands, 72 m elev., 37°31'55"N, 126°21'10"E, Jae Young Park, Nam Kyu Kim, Suldbold Jargalmaa, 26 July 2016, SFC20160726-13 (Holotype, SFC!).

#### Comments.

This species is morphologically similar to *R. mukteshwarica*, but phylogenetically close to *R. pseudoamoenicolor* and *R. pauriensis*. *Russula mukteshwarica* and *R. pauriensis* differ from *Russula* sp. in the greenish yellow region at the pileus center, which is present in the first two species but absent in the latter (Das et al. 2005, 2017). *Russula pseudoamoenicolor* has a discolorous lamella edge (Hyde et al. 2016), whereas *Russula* sp. has a concolorous lamellae edge. *Russula* sp. has a purple pileus similar to that of *R. violeipes*; however, spore ornamentation height (up to 0.7 µm) and pleurocystidia size (80–115 × 12–15 µm) distinguishes *Russula* sp. from *R. violeipes* (Kränzlin 2005).

### Key characters to Asian species in R. subsect. Amoeninae

**Table:** 

1	Basidiome with pinkish pileus	**2**
–	Basidiome with violet or purple pileus	**4**
2	Obtuse hymenial cystidia, subreticulate spore ornamentation, in mixed *Pinus* and *Quercus* forests	***R. bella***
–	Acute hymenial cystidia, subreticulate to reticulate spore ornamentation	**3**
3	Reticulate spore ornamentation, large hymenial cystidia (46‒60 × 9‒12 µm), broad hyphal terminations (6‒9 µm wide) in the pileipellis	***R. punicea***
–	Subreticulate spore ornamentation, small hymenial cystidia (29‒34 × 10‒12.5 µm), narrow hyphal termination (2.5‒4.5 µm wide) in the pileipellis	***R. intervenosa***
4	Reticulate spore ornamentation, pileus with pale cream to yellowish grey color and purplish tinges	***R. orientipurpurea***
–	Subreticulate spore ornamentation, pileus with vivid color	**5**
5	Pileus with violet/greenish/yellowish shades, stipe with purplish flush, large and broad hymenial cystidia	**6**
–	Pileus without greenish or yellowish shades	**7**
6	Small spores with high ornamentation (up to 2 µm), lamellae yellowish, lamellulae absent	***R. pauriensis***
–	Large spores with low ornamentation (up to 0.75 µm), lamellae yellowish to greenish, lamellulae present	***R. mukteshwarica***
7	Lamellar edge discolorous (pastel violet), spore ornamentation up to 2 µm high	***R. pseudoamoenicolor***
–	Lamellar edge concolorous, spore ornamentation up to 1.2 µm high	***Russula* sp.** (SFC20160726-13)

## Discussion

The phylogenetic analyses showed that subsection Amoeninae forms a well-supported monophyletic group. Moreover, the species of Asian, Australian, European, and North American origin form separate clades. Similar results have been reported for other species groups in the various ectomycorrhizal genera of Russulaceae, which are often endemic to specific geographical regions or a single continent (Cho et al. 2018; De Crop et al. 2018; Wang et al. 2018; Lee et al. 2019; Looney et al 2020). Their host plants may have acted as bridges for species dispersal and diversification (Looney et al. 2018), and geographic distance and climate disjunctions may have caused species divergence (Caboň et al. 2019; Lee et al. 2019). The present study included recently obtained sequences of well-known species from different continents except Africa, which will provide useful information for understanding the diversity in this section.

Four *Amoeninae* species had been previously reported from South Korea based on morphological characteristics: two European, one North American, and one Japanese species (Lee et al. 2015). Based on ITS, large ribosomal subunit (LSU), and *rpb2* data, Park et al. (2013) found that the Korean *Amoeninae* species were *R. mariae* (North American) and *R. violeipes* (European). The differences between those results and the present study may be traced back to the limited amount of sequences available at the time. The dramatic increase in the number of sequences available in public databases and the data obtained in the present study allow us to conclude that the species previously identified as *R. mariae* and *R. violeipes* in South Korea are in fact *R. orientipurpurea* and *R. bella*, respectively. We also showed that *R. orientipurpurea* forms a distinct clade that is quite distantly related to North American species, *R. mariae*. Moreover, the color of the pileus and geographical disjunction distinguishes *R. orientipurpurea* from *R. mariae*.

Phylogenetic analysis of LSU sequence data showed a close relationship among *R. bella*, *R. mariae*, and *R. violeipes* (Shimono et al. 2004). Although there are no ITS sequences available of *R. bella* from Japan, one LSU sequence of *R. bella* was identical to that of Korean samples (Park et al. 2013). The European *R. violeipes* is clearly distinguished from *R. bella* (Figs 1, 2). Therefore, considering the available morphological and molecular information, we conclude that all Korean samples previously designated as *R. violeipes* are in fact *R. bella*.

*Russula* sp. (SFC20160726-13) was confirmed to be identical to the three unidentified Chinese samples. The Chinese samples are from Taishan of Shandong Province, which is geographically close to South Korea. They formed a distinct clade and might be a new species. However, there are limited specimens to describe it as a new species. It would be better to introduce this new species after observing more specimens.

The occurrence of a previously reported species from South Korea (Lee et al. 2015), *R. amoena*, was not confirmed in this study. This species was originally described from Europe (Sarnari 1998). Two European specimens of *R. amoena* were included in our phylogenetic analyses, but none of the Korean samples match with these European collections. Thus, so far, *R. amoena* has not been confirmed in South Korea.

Most Korean specimens of *R. bella* and *R. orientipurpurea* were collected from mixed forests with pine and oak trees, which are very common in South Korea. Previous studies have reported that *R. bella* is commonly found as ectomycorrhizal root tips of the conifer species *Abies homolepsis* (Miyamoto et al. 2014), *Pinus amamiana* (Sugiyama et al. 2019), *P. densiflora* (direct GenBank submission), *P. thenderbergii* (Obase et al. 2011; Nakashima et al. 2016), and *P. yunnanensis* (Xie et al. 2010). Some sequences of *R. orientipurpurea* were also obtained from the roots of other *Pinus* spp. (Wen et al. 2015). This indicates that these two species are associated with conifers, but symbiotic relationships with deciduous trees cannot be excluded as the information available in the literature is scarce. *Russula* sp. (SFC20160726-13) seems rare and was collected in a forest dominated by *Quercus mongolica*. Since we have limited sample and data, additional specimens are needed to complete a morphological and ecological characterization of the species. Ecological information can be useful for the identification of morphologically similar ectomycorrhizal fungi species (Nuytinck and Verbeken 2003; Lee et al. 2019). Further investigations focused on ecological information are necessary to obtain a better understanding of the three Korean *Russula* species.

In conclusion, the East Asian Russula species in subsection Amoeninae are distinct from the European and North American species. Three species were identified from South Korea based on molecular and morphological data. However, the molecular data available in GenBank are still limited and comprise only some Russula species in subsection Amoeninae. The amount of ITS data for this group has continuously increased, but protein-coding gene sequences are still insufficient. An overall increase in sequence information would allow for a better understanding of the phylogenetic relationships and global diversity of this group.

## Supplementary Material

XML Treatment for Russula bella

XML Treatment for Russula orientipurpurea

XML Treatment for Russula

## Supplementary materials

Supplementary material 1 Table S1. List of validly published names classified in R. subsect. Amoeninae and allied species around the world Data type species list Explanation note List of validly published names classified in R. subsect. Amoeninae and allied species around the world. https://binary.pensoft.net/file/473301This dataset is made available under the Open Database License (http://opendatacommons.org/licenses/odbl/1.0/). The Open Database License (ODbL) is a license agreement intended to allow users to freely share, modify, and use this Dataset while maintaining this same freedom for others, provided that the original source and author(s) are credited.Komsit Wisitrassameewong, Myung Soo Park, Hyun Lee, Aniket Ghosh, Kanad Das, Bart Buyck, Brian P. Looney, Miroslav Caboň, Slavomír Adamčík, Changmu Kim, Chang Sun Kim, Young Woon Lim

Supplementary material 2 Table S2. Sequences used for the ITS analyses in this study Data type ITS sequences Explanation note Sequences used for the ITS analyses in this study. ITS sequences generated in this study are presented in boldface. (T) indicates the type specimen. Species names in bracket are the original species epithet in GenBank or Park et al. (2013). https://binary.pensoft.net/file/473302This dataset is made available under the Open Database License (http://opendatacommons.org/licenses/odbl/1.0/). The Open Database License (ODbL) is a license agreement intended to allow users to freely share, modify, and use this Dataset while maintaining this same freedom for others, provided that the original source and author(s) are credited.Komsit Wisitrassameewong, Myung Soo Park, Hyun Lee, Aniket Ghosh, Kanad Das, Bart Buyck, Brian P. Looney, Miroslav Caboň, Slavomír Adamčík, Changmu Kim, Chang Sun Kim, Young Woon Lim
