# Supplementary material for: Taxonomic revision of Russula subsection Amoeninae from South Korea
Source: MycoKeys. 2020 Nov 9;75:1–29. doi: 10.3897/mycokeys.75.53673 (PMC7669817; doi:10.3897/mycokeys.75.53673)
Supplement: Supplementary material 1 — Table S1. List of validly published names classified in R. subsect. Amoeninae and allied species around the world [file mycokeys-75-001-s001.docx]

**Supplementary Table 1:** List of validly published names classified in *R.* subsect. *Amoeninae* and allied species around the world

| **Species** | **Distribution** | **Relevant literature** |
| --- | --- | --- |
| *R. aciculocystis* Kauffman ex Bill & O.K. Mill. | USA | Bills and Miller 1984 |
| *R. alachuana* Murrill | USA | Looney 2015 |
| *R. amoena* f. *acystidiata* Romagn. | Europe | Romagnesi 1985, Sarnari 1998 |
| *R. amoena* Quél. | Europe | Quélet 1880, Sarnari 1998 |
| *R. amoenicolor* f. *olivacea* (Maire) Singer | Europe | Singer 1932, Sarnari 1998 |
| *R. amoenicolor* Romagn. | Europe | Romagnesi 1962, Sarnari 1998 |
| *R. annulatobadia* Beeli | Rep. Dem. Congo | Beeli 1936 |
| *R. bella* Hongo | Japan | Hongo 1968 |
| *R. bonii* Buyck | Zambia | Buyck 1995 |
| *R. ciliata* Buyck | Zambia, Zimbabwe, Tanzania | Buyck 1987, Buyck 1995, Härk√nen et al. 1993, Buyck & Sharp 2007 |
| *R. diversicolor* Pegler | Martinique | Buyck 1992 |
| *R. epitheliosa* Singer | Brazil | Buyck 1992 |
| *R. fimbriata* Buyck | Rep .Dem. Congo | Buyck 1987 |
| *R. granulosula* Murrill | USA | Kibby & Fatto 1900 |
| *R. hibbardae* Burl. | USA | Singer 1975 |
| *R. incrassata* Mussat | Rep .Dem. Congo | Buyck 1987 |
| *R. intervenosa* S. Paloi, A.K. Dutta & K. Acharya | India | Crous et al. 2016 |
| *R. madecassensis* R. Heim. | Madagascar | Heim 1938 |
| *R. mariae* Peck | USA | Peck 1872, Adamčík et al. 2018 |
| *R. mukteswarica* K. Das, S.L. Mill., J.R. Sharma & R.P. Bhatt | India | Das et al. 2005 |
| *R. pauriensis* A. Ghosh, K. Das & Buyck | India | Das et al. 2017 |
| *R. pausiaca* Buyck | Rep .Dem. Congo | Buyck 1987 |
| *R. praeformosa* Murrill | USA | Singer 1975 |
| *R. pseudoamoenicolor* A. Ghosh, Buyck, K. Das, A. Baghela & Bhatt | India | Hyde et al. 2016 |
| *R. punicea* Chiu | China | Chiu 1945 |
| *R. rostraticystidiata* T. Lebel | Australia | Lebel & Tonkin 2007 |
| *R. smithii* Singer | USA | Singer 1942b |
| *R. subcyanoxantha* Murrill | USA | Kibby and Fatto 1990 |
| *R. subincarnata* Murrill | USA | Murrill 1941, Singer 1958 |
| *R. subviridella* Murrill | USA | Murrill 1943, Kibby and Fatto 1990 |
| *R. sulcatipes* Murrill | USA | Singer 1942a |
| *R. tuberculata* Murrill | USA | Murrill 1940 |
| *R. variegata* Romagn | USA | Singer 1975 |
| *R. variicolor* Murrill | USA | Looney 2015 |
| *R. variispora* T. Lebel | Australia | Lebel & Tonkin 2007 |
| *R. violeipes* f. *citrina* (Quél.) Maire | Europe | Maire 1910, Sarnari 1998 |
| *R. violeipes* Quél. | Europe | Quélet 1898, Sarnari 1998 |
