# Supplementary material for: Taxonomic revision of Russula subsection Amoeninae from South Korea
Source: MycoKeys. 2020 Nov 9;75:1–29. doi: 10.3897/mycokeys.75.53673 (PMC7669817; doi:10.3897/mycokeys.75.53673)
Supplement: Supplementary material 2 — Table S2. Sequences used for the ITS analyses in this study [file mycokeys-75-001-s002.docx]

**Supplementary table2:** Sequences used for the ITS analyses in this study. ITS sequences generated in this study are presented in boldface. (T) indicates the type specimen. Species names in bracket are the original species epithet in GenBank or Park et al. (2013)

| **Taxon** | **Herbarium no./clone** | **Locality** | **GenBank no.** | **Source** |
| --- | --- | --- | --- | --- |
| Outgroup | | | | |
| *R. grisea* | FH12-234 | Germany | KT934006 | Fruiting body |
| *R. virescens* | HJB9989 | Belgium | DQ422014 | Fruiting body |
| Subsect. *Amoeninae* | | | | |
| *Macowanites* sp. | F33_12 | Australia | KY697596 | Fruiting body |
| ***R. alachuana*** | **SAV F-20113** | **USA** | **MT017542** | **Fruiting body** |
| ***R. alachuana*** | **SAV F-20108** | **USA** | **MT017543** | **Fruiting body** |
| *R. alachuana* (*Russula* sp.) | S.D. Russell MycoMap 6332 | USA | MK532805 | Fruiting body |
| *R. alachuana* (*Russula* sp.) | 17 | USA | HQ667813 | Roots of *Hexalectis brevicaulis* |
| *R. alachuana* (*Russula* sp.) | FLAS-F-60871 | USA | MH016858 | Fruiting body |
| *R. alachuana* (*Russula* sp.) | FLAS-F-61265 | USA | MH211844 | Fruiting body |
| *R. alachuana* (*Russula.* sp.) | FLAS-F-61313 | USA | MH211876 | Fruiting body |
| ***R. amoena*** | **SAV F-1352** | **Slovakia** | **MT017545** | **Fruiting body** |
| ***R. amoena*** | **SAV F-3147** | **Slovakia** | **MT017544** | **Fruiting body** |
| *R. amoenicolor* | 311IX76 |  | AY061655 | Fruiting body |
| *R. andaluciana* | BM360 (T) | Spain | MK105627 | Fruiting body |
| *R. andaluciana* | 39198 (AH) | Spain | MK105628 | Fruiting body |
| ***R.* cf. *amoenicolor*** | **SAV F-20302** | **Greece** | **MT017546** | **Fruiting body** |
| ***R.* cf. *amoenicolor*** | **SAV F-20324** | **Greece** | **MT017547** | **Fruiting body** |
| ***R. bella*** | **SFC20170819-05** | **South Korea** | **MT017552** | **Fruiting body** |
| ***R. bella*** | **SFC20120722-03** | **South Korea** | **MT017553** | **Fruiting body** |
| ***R. bella*** | **HCCN15410** | **South Korea** | **MT017554** | **Fruiting body** |
| ***R. bella*** | **SFC20170819-10** | **South Korea** | **MT017555** | **Fruiting body** |
| ***R. bella*** | **SFC20170731-02** | **South Korea** | **MT017556** | **Fruiting body** |
| *R. bella* (*Russula* sp.) | A2-2-34 | Japan | LC096290 | Root of *Pyrola japonica* |
| *R. bella* (*Russula* sp.) | AQ1-1-13 | Japan | LC096398 | Root of *Pyrola japonica* |
| *R. bella* (*Russula* sp.) | A2-2-7 | Japan | LC096297 | Root of *Pyrola japonica* |
| *R. bella* (*Russula* sp.) | AP1-3-2 | Japan | LC096381 | Root of *Pyrola japonica* |
| *R. bella* (*Russula* sp.) | A2-1-20 | Japan | LC096273 | Root of *Pyrola japonica* |
| *R. bella* (*Russula* sp.) | AP1-3-51 | Japan | LC096391 | Root of *Pyrola japonica* |
| *R. bella* (*Russula* sp.) | AP1-3-9 | Japan | LC096395 | Root of *Pyrola japonica* |
| *R. bella* (*Russula* sp.) | AP1-3-37 | Japan | LC096387 | Root of *Pyrola japonica* |
| *R. bella* (*Russula* sp.) | AP1-3-1 | Japan | LC096377 | Root of *Pyrola japonica* |
| *R. bella* (*Russula* sp.) | A2-1-46 | Japan | LC096279 | Root of *Pyrola japonica* |
| *R. bella* (*Russula* sp.) | A2-1-23 | Japan | LC096275 | Root of *Pyrola japonica* |
| *R. bella* (*Russula* sp.) | AP1-3-7 | Japan | LC096394 | Root of *Pyrola japonica* |
| *R. bella* (*Russula* sp.) | A2-2-55 | Japan | LC096296 | Root of *Pyrola japonica* |
| *R. bella* (*Russula* sp.) | A2-2-20 | Japan | LC096288 | Root of *Pyrola japonica* |
| *R. bella* (*Russula* sp.) | A2-2-37 | Japan | LC096293 | Root of *Pyrola japonica* |
| *R. bella* (*Russula* sp.) | AP1-3-21 | Japan | LC096382 | Root of *Pyrola japonica* |
| *R. bella* (*Russula* sp.) | AP1-3-35 | Japan | LC096385 | Root of *Pyrola japonica* |
| *R. bella* (*Russula* sp.) | AP1-3-48 | Japan | LC096388 | Root of *Pyrola japonica* |
| *R. bella* (*Russula* sp.) | AP1-3-17 | Japan | LC096380 | Root of *Pyrola japonica* |
| *R. bella* (*Russula* sp.) | A2-1-45 | Japan | LC096278 | Root of *Pyrola japonica* |
| *R. bella* (*Russula* sp.) | I-1 | South Korea | AB571503 | Root of *Pinus densiflora* |
| *R. bella* (*Russula* sp.) | P09145 | South Korea | AB587767 | Root of *Pinus thunbergii* |
| *R. bella* (*Russula* sp.) | ys50 | Japan | LC364244 | Root of *Pinus amamiana* |
| *R. bella* (*Russula* sp.) | YM1184 | Japan | AB848571 | Root of *Abies homolepis* |
| *R. bella* (*Russula* sp.) |  | Japan | LC033912 | Root of *Pinus thunbergii* |
| *R. bella* (*Russula* sp.) | 68-2A | China | JQ396501 | Root of *Monotropa hypopithys* |
| *R. bella* (*R.* cfr. *violeipes*) | A0543 | China | GU371290 | Root of *Pinus yunnanensis* |
| *R. bella* (*Russula* sp.) | HMAS:270976 | China | KX441175 | Fruiting body |
| *R. bella* (*Russula* sp.) | HMAS:270985 | China | KX441180 | Fruiting body |
| *R. bella* (*Russula* sp.) | HMAS:270981 | China | KX441177 | Fruiting body |
| *R. bella* (*Russula* sp.) | HMAS:280183 | China | LT602954 | Fruiting body |
| *R. bella* (*Russula* sp.) | HMAS:265019 | China | KX441088 | Fruiting body |
| *R. bella* (*Russula* sp.) | 170728F8 | China | MK748184 | Fruiting body |
| *R. bella* (*Russula* sp.) | 180824H3 | China | MK748182 | Fruiting body |
| *R. bella* (*Russula* sp.) | HMAS:271043 | China | KX441190 | Fruiting body |
| *R. bella* (*Russula* sp.) | KN-RMA | Japan | KJ482564 | Fruiting body |
| *R. bella* (*R. violeipes*) | SFC20120915-16 | South Korea | KF361802 | Fruiting body |
| *R. bella* (*R. violeipes*) | SFC20120919-27 | South Korea | KF361803 | Fruiting body |
| *R. bella* (*R. violeipes*) | HCCN16435 | South Korea | KF361781 | Fruiting body |
| *R. bella* (*R. violeipes*) | SFC20120704-32 | South Korea | KF361790 | Fruiting body |
| *R. bella* (*R. violeipes*) | HCCN22654 | South Korea | KF361788 | Fruiting body |
| *R. bella* (*R. violeipes*) | SFC20120727-05 | South Korea | KF361793 | Fruiting body |
| *R. bella* (*R. violeipes*) | SFC20120731-06 | South Korea | KF361796 | Fruiting body |
| *R. bella* (*R. violeipes*) | SFC20120919-51 | South Korea | KF361805 | Fruiting body |
| *R. bella* (*R. violeipes*) | SFC20120726-33 | South Korea | KF361792 | Fruiting body |
| *R. bella* (*R. violeipes*) | SFC20120817-08 | South Korea | KF361798 | Fruiting body |
| *R. bella* (*R. violeipes*) | SFC20120817-09 | South Korea | KF361799 | Fruiting body |
| *R. bella* (*R. violeipes*) | HCCN16735 | South Korea | KF361783 | Fruiting body |
| *R. bella* (*R. violeipes*) | SFC20120731-02 | South Korea | KF361795 | Fruiting body |
| *R. bella* (*R. violeipes*) | SFC20120814-23 | South Korea | KF361797 | Fruiting body |
| *R. bella* (*R. violeipes*) | KA13-1196 | South Korea | KR673669 | Fruiting body |
| *R. bella* (*R. violeipes*) | HCCN21655 | South Korea | KF361786 | Fruiting body |
| *R. bella* (*R. violeipes*) | HCCN11773 | South Korea | KF361780 | Fruiting body |
| *R. bella* (*R. violeipes*) | HCCN16818 | South Korea | KF361784 | Fruiting body |
| *R. bella* (*R. violeipes*) | SFC20120905-07 | South Korea | KF361801 | Fruiting body |
| *R. bella* (*R. violeipes*) | SFC20120821-71 | South Korea | KF361800 | Fruiting body |
| *R. bella* (*R. violeipes*) | HCCN23030 | South Korea | KF361789 | Fruiting body |
| *R. bella* (*R. violeipes*) | SFC20120731-01 | South Korea | KF361794 | Fruiting body |
| *R. bella* (*R. violeipes*) | HCCN16459 | South Korea | KF361782 | Fruiting body |
| *R. bella* (*R. violeipes*) | SFC20121010-06 | South Korea | KF361808 | Fruiting body |
| *R. bella* (*R. violeipes*) | HCCN21858 | South Korea | KF361787 | Fruiting body |
| *R. bella* (*R. violeipes*) | SFC20120719-04 | South Korea | KF361791 | Fruiting body |
| *R. bella* (*R. violeipes*) | HCCN10627 | South Korea | KF361779 | Fruiting body |
| *R. bella* (*R. violeipes*) | SFC20120929-05 | South Korea | KF361806 | Fruiting body |
| *R. bella* (*R. violeipes*) | SFC20121005-09 | South Korea | KF361807 | Fruiting body |
| *R. bella* (*R. violeipes*) | SFC20120919-29 | South Korea | KF361804 | Fruiting body |
| *R. bella* (*R. violeipes*) | HCCN20149 | South Korea | KF361785 | Fruiting body |
| *R. intervenosa* | CUH AM273 (T) | India | MK414579 | Fruiting body |
| ***R. mariae*** | **SAV F-4484** | **USA** | **MT017557** | **Fruiting body** |
| ***R. mariae*** | **SAV F-4493** | **USA** | **MT017558** | **Fruiting body** |
| ***R. mariae*** | **SAV F-4564** | **USA** | **MT017559** | **Fruiting body** |
| *R. mariae* | JMP0063 | USA | EU819426 | Fruiting body |
| *R. mariae* | PC BB2004-213 | USA | EU598199 | Fruiting body |
| *R. mariae* | BHI-F229a | USA | MF161211 | Fruiting body |
| *R. mariae* | FH:BHI-F439 | USA | MF161267 | Soil |
| *R. mariae* (*R.* aff. *mariae*) | L2Z_4_11 | USA | JX030253 | Root of *Castanea dendata* |
| *R. mariae* (*Russula* sp.) | B06T1-68-2 | USA | FM999501 | Fruiting body |
| *R. mariae* (*Russula* sp.) | BC063531-23 | USA | FM999598 | Soil |
| *R. mariae* (*Russula* sp.) | BC063531-7 | USA | FM999589 | Soil |
| *R. mariae* (*Russula* sp.) | BHI-F429a | USA | MF161263 | Fruiting body |
| *R. mariae* (*Russula* sp.) | BC063531-27 | USA | FM999600 | Soil |
| *R. mariae* (*Russula* sp.) |  | USA | GU907783 | Roots of *Quercus rubra* |
| *R. mariae* (*Russula* sp.) | S0744 | USA | FM999622 | Fruiting body |
| *R. nitida* | PC BB2004-272 | USA | EU598164 | Fruiting body |
| ***R. orientipurpurea*** | **SFC20170725-37 (T)** | **South Korea** | **MT017548** | **Fruiting body** |
| ***R. orientipurpurea*** | **SFC20170821-22b** | **South Korea** | **MT017549** | **Fruiting body** |
| ***R. orientipurpurea*** | **SFC20170819-08** | **South Korea** | **MT017550** | **Fruiting body** |
| ***R. orientipurpurea*** | **SFC20170726-47** | **South Korea** | **MT017551** | **Fruiting body** |
| *R. orientipurpurea* (*R. mariae*) | SFC20120724-23 | South Korea | KF361768 | Fruiting body |
| *R. orientipurpurea* (*R. mariae*) | HCCN23016 | South Korea | KF361766 | Fruiting body |
| *R. orientipurpurea* (*R. mariae*) | SFC20120825-02 | South Korea | KF361773 | Fruiting body |
| *R. orientipurpurea* (*R. mariae*) | HMJAU 27831 | China | KY681436 | Fruiting body |
| *R. orientipurpurea* (*R. mariae*) | SFC20120919-37 | South Korea | KF361776 | Fruiting body |
| *R. orientipurpurea* (*R. mariae*) | HCCN18800 | South Korea | KF361761 | Fruiting body |
| *R. orientipurpurea* (*R. mariae*) | SFC20120831-04 | South Korea | KF361774 | Fruiting body |
| *R. orientipurpurea* (*R. mariae*) | SFC20120922-08 | South Korea | KF361778 | Fruiting body |
| *R. orientipurpurea* (*R. mariae*) | SFC20120804-09 | South Korea | KF361771 | Fruiting body |
| *R. orientipurpurea* (*R. mariae*) | SFC20120922-07 | South Korea | KF361777 | Fruiting body |
| *R. orientipurpurea* (*R. mariae*) | HCCN23379 | South Korea | KF361767 | Fruiting body |
| *R. orientipurpurea* (*R. mariae*) | HCCN16834 | South Korea | KF361759 | Fruiting body |
| *R. orientipurpurea* (*R. mariae*) | SFC20120821-39 | South Korea | KF361772 | Fruiting body |
| *R. orientipurpurea* (*R. mariae*) | SFC20120915-10 | South Korea | KF361775 | Fruiting body |
| *R. orientipurpurea* (*R. mariae*) | SFC20120726-37 | South Korea | KF361770 | Fruiting body |
| *R. orientipurpurea* (*R. mariae*) | SFC20120725-45 | South Korea | KF361769 | Fruiting body |
| *R. orientipurpurea* (*R. mariae*) | HCCN22939 | South Korea | KF361765 | Fruiting body |
| *R. orientipurpurea* (*R. mariae*) | HCCN20514 | South Korea | KF361763 | Fruiting body |
| *R. orientipurpurea* (*R. mariae*) | ASIS26883 | South Korea | KU139525 | Fruiting body |
| *R. orientipurpurea* (*R. mariae*) | HCCN18725 | South Korea | KF361760 | Fruiting body |
| *R. orientipurpurea* (*R. mariae*) | HCCN21685 | South Korea | KF361764 | Fruiting body |
| *R. orientipurpurea* (*R. mariae*) | HCCN19111 | South Korea | KF361762 | Fruiting body |
| *R. orientipurpurea* (*Russula* sp.) | MAS-2010 | Japan | GQ359821 | Fruiting body |
| *R. orientipurpurea* (*Russula* sp.) | ECM184 | China | JQ991809 | Ectomycorrhizal root tips |
| *R. orientipurpurea* (*Russula* sp.) | HMAS:263063 | China | KX441071 | Fruiting body |
| *R. orientipurpurea* (*Russula* sp.) | EMF60 | China | JF273558 | Fruiting body |
| *R. orientipurpurea (Russula* sp.) | Pj1-mOTU053 | China | AB982036 | Ectomycorrhizal root tips |
| *R. orientipurpurea (Russula* sp.) | Pj1-mOTU055 | China | AB982038 | Roots of *Pinus massoniana* |
| *R. orientipurpurea (Russula* sp.) | KN_RVI1 | Japan | KJ482566 | Fruiting body |
| *R. rostraticystidia* | H6165 (T) | Australia | EU019938 | Fruiting body |
| *R. pauriensis* | CAL:1395 (T) | India | MF535185 | Fruiting body |
| *R. pseudoamoenicolor* | AG 15-739 | India | KX234819 | Fruiting body |
| *R. variispora* | H5855(T) | Australia | EU019934 | Fruiting body |
| *R. violeipes* | 108 | Spain | KY693648 | Roots of *Pinus pinaster* |
| *R. violeipes* | 208IS76 |  | AY061726 | Fruiting body |
| *R. violeipes* | 1792 | Italy | JF908655 | Fruiting body |
| *R. violeipes* (*Russula.* sp.) | HA-A12 | Switzerland | KX886145 | Roots of *Fagus sylvatica* |
| ***Russula* sp.** | **SAV F-4063** | **USA** | **MT017560** | **Fruiting body** |
| ***Russula* sp.** | **SAV F-20117** | **USA** | **MT017562** | **Fruiting body** |
| ***Russula* sp.** | **SAV F-20134** | **USA** | **MT017563** | **Fruiting body** |
| *Russula*. sp. | FP3-D9 | USA | MF946125 | Ectomycorrhizal roots |
| *Russula*. sp. | SC90 | USA | AJ633583 | Roots of *Pinus taeda* |
| *Russula*. sp. | SC69 | USA | AJ633577 | Roots of *Pinus taeda* |
| *Russula*. sp. | SC66 | USA | AJ633575 | Roots of *Pinus taeda* |
| *Russula*. sp. | FP18-B5 | USA | MF946183 | Ectomycorrhizal roots |
| *Russula*. sp. | 24B_G1_E11 | USA | KX899245 | Roots of *Pinus clausa* |
| *Russula*. sp. | FLAS-F-61711 | USA | MH212063 | Fruiting body |
| *Russula*. sp. | FP59-C3 | USA | MF946235 | Ectomycorrhizal roots |
| *Russula*. sp. | FP14-D3 | USA | MF946095 | Ectomycorrhizal roots |
| *Russula*. sp. | FP7-G3 | USA | MF946277 | Ectomycorrhizal roots |
| *Russula*. sp. | FLAS-F-61622 | USA | MH212005 | Fruiting body |
| *Russula*. sp. | 31B_T1_D12 | USA | KX899417 | Roots of *Pinus clausa* |
| *Russula*. sp. | T998 | Australia | JF960823 | Fruiting body |
| *Russula*. sp. | HMAS:268959 | China | KX441127 | Fruiting body |
| *Russula*. sp. | 180824H21 | China | MK748183 | Fruiting body |
| *Russula*. sp. | 180717C17 | China | MK748180 | Fruiting body |
| *Russula*. sp. | 180717C9 | China | MK748181 | Fruiting body |
| *Russula*. sp. | BAAC125 | Panama | KM595029 | Roots of *Oreomunnea mexicana* |
| *Russula*. sp. | R75 | Spain | JQ975980 | Roots of *Pinus pinaster* |
| *Russula*. sp. | FLAS-F-61195 | USA | MH211804 | Fruiting body |
| *Russula*. sp. | Rus1 | Thailand | AB854679 | Fruiting body |
| *Russula*. sp. | CUB:Microbiology KHS5 | Thailand | AB459514 | Fruiting body |
| *Russula*. sp. | 02DBH070700077 | France | FN610993 | Soil from *Fagus sylvatica* |
| *Russula*. sp. | 02DBH070700024 | France | FN610951 | Soil from *Fagus sylvatica* |
| ***Russula* sp.** | **SFC20160726-13** | **South Korea** | **MT017561** | **Fruiting body** |
